# Dynamic Intermediate-Temperature CO_2_ Adsorption Performance of K_2_CO_3_-Promoted Layered Double Hydroxide-Derived Adsorbents

**DOI:** 10.3390/molecules29061192

**Published:** 2024-03-07

**Authors:** Ruotong Li, Xixuan Hu, Liang Huang, Nicholas Mulei Musyoka, Tianshan Xue, Qiang Wang

**Affiliations:** 1 Engineering Research Center for Water Pollution Source Control & Eco-Remediation, College of Environmental Science and Engineering, Beijing Forestry University, Beijing 100083, China; 2 State Key Laboratory of Efficient Production of Forest Resources, Beijing Forestry University, Beijing 100083, China; 3 Nottingham Ningbo China Beacons of Excellence Research and Innovation Institute, University of Nottingham Ningbo China, Ningbo 315100, China; 4 Institute of Atmospheric Environment, Chinese Research Academy of Environmental Sciences, Beijing 100012, China

**Keywords:** K_2_CO_3_-promoted LDHs, organic anion intercalation, inorganic anion intercalation, CO_2_ dynamic adsorption, adsorption capacity, intermediate-temperature

## Abstract

The dynamic adsorption characteristics of K_2_CO_3_-promoted layered double hydroxides (LDHs)-based adsorbent, with organic and inorganic anion intercalation, were studied. MgAl–LDH, K_2_CO_3_/MgAl–LDH, and K_2_CO_3_/MgAl–LDH(C16) with varying K_2_CO_3_ loads were prepared and used for intermediate-temperature CO_2_ sequestration. The adsorbent was thoroughly characterized using X-ray diffraction, Brunauer–Emmett–Teller, scanning electron microscopy, and Fourier Transform Infrared Spectroscopy techniques, which revealed enhanced adsorption properties of MgAl–LDH, due to K_2_CO_3_ promotion. Thermogravimetric CO_2_ adsorption tests on the constructed adsorbent materials showed that the 12.5 wt% K_2_CO_3_/MgAl–LDH(C16) adsorbent with organic anion intercalation exhibited optimal adsorption activity, achieving an adsorption capacity of 1.12 mmol/g at 100% CO_2_ and 350 °C. However, fixed-bed dynamic adsorption tests yielded different results; the 25 wt% K_2_CO_3_/MgAl–LDH prepared through inorganic anion intercalation exhibited the best adsorption performance in low-concentration CO_2_ penetration tests. The recorded penetration time was 93.1 s, accompanied by an adsorption capacity of 0.722 mmol/g. This can be attributed to the faster adsorption kinetics exhibited by the 25 wt% K_2_CO_3_/MgAl–LDH adsorbent during the early stages of adsorption, thereby facilitating efficient CO_2_ capture in low-concentration CO_2_ streams. This is a conclusion that differs from previous reports. Earlier reports indicated that LDHs with organic anion intercalation exhibited higher CO_2_ adsorption activity in thermogravimetric analyzer tests. However, this study found that for the fixed-bed dynamic adsorption process, K_2_CO_3_-modified inorganic anion-intercalated LDHs perform better, indicating their greater potential in practical applications.

## 1. Introduction

The substantial emissions of greenhouse gases (GHGs) contribute significantly to escalating global temperatures and climate deterioration, with CO_2_ being the primary greenhouse gas, responsible for approximately 66% of global warming. Various human activities, including the burning of fossil fuels such as coal, oil, and natural gas, as well as agricultural practices, drive an accelerated surge in CO_2_ levels [1,2]. These activities trigger a spectrum of extreme weather events, including climate change, sea level rise, land desertification, and agricultural decline. The exploration of CO_2_ capture, utilization, and storage (CCUS) is as a crucial approach to curbing CO_2_ emissions and represents a pivotal technology for carbon emission mitigation [3,4]. CCUS technology finds application primarily in traditional oil displacement and chemical utilization, exhibiting promising prospects for development [5,6]. 

Among various carbon reduction technologies, the sorption-enhanced water gas shift (SEWGS) is a pre-combustion CO_2_ capture technology that combines water gas shift (WGS) and adsorbed CO_2_ within a single container, achieving both high CO conversion and substantial CO_2_ recovery [7,8,9]. The WGS process converts CO and H_2_O into CO_2_ and H_2_, while SEWGS manipulates Le Chatelier’s principle to shift the thermodynamic equilibrium towards H_2_ production, streamlining the process and enhancing energy efficiency [10,11]. The temperature range conducive to the water–gas shift reaction typically spans from 200 °C to 400 °C. Within this range, CO_2_ adsorption materials exhibiting commendable performance encompass K_2_CO_3_-promoted LDHs, Dawsonite and MgO-based adsorbents [12]. Studies have been conducted to achieve the production of high-purity H_2_ by combining these two medium-temperature CO_2_ adsorbents for the SEWGS reaction carried out in a multi-layer reactor, which can be stable over 10 cycles with a long duration [13]. Dawsonite is also a promising adsorbent in the SEWGS process, with a moderate adsorption capacity (0.3–0.7 mmol/g) and a fast adsorption rate, achieving 90% absorption in 15–20 min [14]. Additionally, loading K_2_CO_3_ onto MgO-based adsorbents is another method to increase the adsorption capacity [15]. K_2_CO_3_-promoted LDH adsorbents showcase both high and consistent CO_2_ adsorption capacities, coupled with rapid CO_2_ adsorption kinetics at moderate temperatures, rendering them one of the most promising adsorbents for the SEWGS process [16,17]. 

LDHs are a class of materials consisting of a positively charged bivalent metal ion layer and a negatively charged anion layer [18,19,20]. An LDH has a unique two-dimensional (2D) layered structure that facilitates facile alteration of anions and cations, alongside a memory effect. These attributes have led to extensive research in catalytic and environmental domains, finding applications in adsorption, oxygen evolution, wastewater treatment, and CO_2_ reduction, among other areas [21,22,23,24]. Moreover, LDH synthesis methods are relatively straightforward, encompassing the co-precipitation method, ion exchange method, hydrothermal method, sol-gel method, and more [17,25,26,27,28]. LDH is considered to be a material with high low-concentration CO_2_ capture activity in a medium temperature range of 200–450 °C, and the factors affecting its performance include Mg/Al ratio, surface modification, etc. [29]. The performance of an LDH can be improved by adding potassium (K_2_CO_3_ as the source) [30,31]. In addition to LDHs, MgO-based adsorbents are also one of the commonly used adsorbents in intermediate-temperature CO_2_ capture [32]. Despite the high theoretical adsorption capacity of MgO-based adsorbents, the bulk MgO exhibits sluggish kinetic reactivity, resulting in a very low CO_2_ capture capacity of less than 0.1 mmol/g [33,34,35]. The CO_2_ capture capabilities of MgO-based adsorbents, when promoted by alkali nitrate mixtures, have seen substantial enhancement, achieving a noteworthy 19.06 mmol/g at 325 °C [36,37]. However, subsequent studies reveal that the CO_2_ adsorption performance of molten salt-modified MgO materials is notably influenced by CO_2_ concentration. Specifically, CO_2_ with a concentration below 20% is almost unable to be adsorbed by MgO, thereby impacting the practical viability of MgO in CO_2_ separation processes.

Compared to MgO, the issue with LDH adsorbents lies in their lower adsorption capacity. Traditionally reported LDH adsorbents exhibit an adsorption capacity around 0.5 mmol/g, and, even after modification, this figure only increases to about 1 mmol/g. This limitation poses a challenge to the utilization of LDH. To address these issues, Wang et al. [38] embedded long carbon chain organic anions into LDH, resulting in a significant improvement in CO_2_ capture capacity up to 1.25 mmol/g. The organic anions with extended carbon chains will produce large amounts of gaseous byproducts, such as CO_2_ and H_2_O, during the decomposition process, which is conducive to the formation of micropores in the mixed oxides and to improving the CO_2_ capture capacity. Li et al. [39] used K_2_CO_3_ to promote LDHs as a precursor for the pre-combustion of a CO_2_ adsorbent with a CO_2_ capacity of up to 1.93 mmol/g at 300 °C. Qin et al. [40] studied the promoting effect of carbon chain length of carboxylic acid anion on CO_2_ capture performance of LDH. The results show that CO_2_ capture capacity increases with the increase in carbon chain length. By coating with 55 mol% (Li-Na-K)NO_3_ molten salt, the CO_2_ capture capacity can be increased to 3.25 mmol/g. However, all these assessments were conducted using thermogravimetric analyzer (TGA), which only evaluates the increase in weight of the adsorbent material in a CO_2_ atmosphere. It does not comprehensively reflect the adsorbent’s actual performance in terms of CO_2_ removal efficiency, breakthrough time, and dynamic adsorption capacity in real-life scenarios. Therefore, we performed dynamic adsorption tests on the adsorbent materials in a simulated application setting within a fixed bed.

In this contribution, the dynamic adsorption characteristics of K_2_CO_3_-promoted LDHs at intermediate temperature were systematically studied, and the properties of organic anion- and inorganic anion-intercalated LDH in TGA and fixed beds were compared. Comprehensive characterization was performed on all synthesized LDH materials and the impact of CO_2_ concentration and K_2_CO_3_ loading on CO_2_ capture capacity were studied. Additionally, an analysis was conducted to elucidate the differences observed between the optimal adsorbent in TGA adsorption and dynamic adsorption scenarios.

## 2. Results and Discussion

### 2.1. The CO_2_ Capture Performance of Layered Double Hydroxides (LDH) and MgO Promoted with Molten Salt

**Figure 1:**
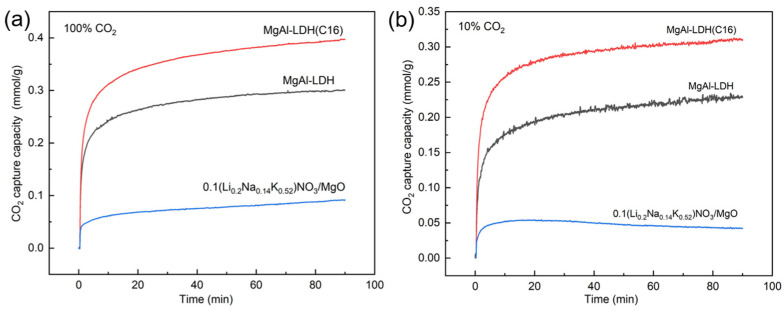
CO_2_ adsorption capacity of LDH and modified MgO at (**a**) 100% CO_2_ and (**b**) 10% CO_2_.

The intermediate-temperature CO_2_ adsorption can generally be applied to the WGS reaction or the CO_2_ removal from flue gas, with temperatures typically ranging from 200–400 °C. At this temperature, commonly used adsorbents include MgO-based adsorbents and LDH-based adsorbents. For the WGS reaction, a lower temperature is favorable for promoting hydrogen production, but it also results in slow reaction rates. Considering practical application scenarios, the selected temperature for this study was 350 °C. MgAl–LDH, MgAl–LDH(C16), and molten salt-promoted MgO were synthesized for CO_2_ adsorption. Figure 1a shows the adsorption properties of LDH and MgO at high concentrations of CO_2_. The adsorption capacity of an organic anion intercalation LDH was found to be superior to that of inorganic carbonate ion intercalation, with MgAl–LDH(C16) achieving a capacity of 0.4 mmol/g at 100% CO_2_. Figure 1b presents the adsorption performance at low CO_2_ concentration, where several materials exhibited a similar trend to high concentration adsorption. MgAl–LDH(C16) achieved a capacity of 0.3 mmol/g at 10% CO_2_.

As reported in the literature, MgO promoted with molten salt is commonly used for CO_2_ adsorption at intermediate temperatures and exhibits favorable adsorption effects. Figure 1 illustrates the adsorption capacities of MgO under both high and low CO_2_ concentration conditions. At 350 °C, the adsorption capacity of molten salt-promoted MgO was observed to be very low for both high and low CO_2_ concentrations. However, the previous literature suggests that modified MgO can achieve an adsorption capacity of 12 mmol/g at 300 °C [41]. Hence, variation in temperature significantly influences the adsorption capacity of MgO, while LDHs demonstrate better performance and faster adsorption rates at 350 °C. Therefore, this paper will not further explore modified MgO.

**Figure 2:**
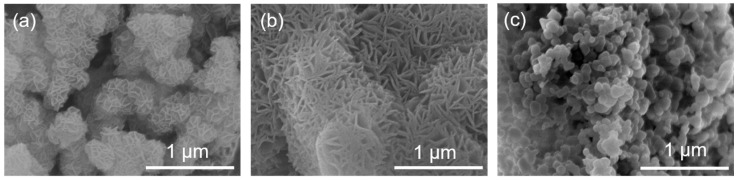
SEM images of (**a**) MgAl–LDH, (**b**) MgAl–LDH(C16), and (**c**) 0.1(Li_0.2_Na_0.14_K_0.52_)NO_3_/MgO.

The scanning electron microscopy (SEM) analysis was conducted to examine the morphologies of synthesized samples, and Figure 2 illustrates the morphologies and microstructure of MgAl–LDH, MgAl–LDH(C16), and modified MgO. The results show that after calcination at 400 °C, the LDH’s plate form was decomposed into nanoparticles [42]. MgAl–LDH exhibited a lamellar accumulation structure, whereas MgAl–LDH(C16) remained as lamellar nanoparticles even after calcination. Both LDHs exhibited a rose-like appearance, which is the result of selective growth along the (001) surface. In contrast, modified MgO exhibited a small spherical particle structure, which differed significantly from the sheet structure of the LDH. Furthermore, a N_2_ adsorption–desorption analysis was performed to evaluate the specific surface area and pore structure of the calcined LDH, as present in Figure S1. According to the International Union of Pure and Applied Chemistry (IUPAC) classification, the samples showed a type IV isotherm and a type H3 hysteresis loop, indicating that there were slit holes and channels. The H3 ring is thought to be related to plate aggregation. The specific surface area was calculated using the Brunauer–Emmett–Teller (BET) equation and the pore volume distribution was calculated using the Barrett–Joyner–Halenda (BJH) method [43]. The analysis revealed that the specific surface area (SSA) of molten salt-promoted MgO is smaller, almost ten times less, compared to that of LDH, as shown in Table 1.

**Figure 3:**
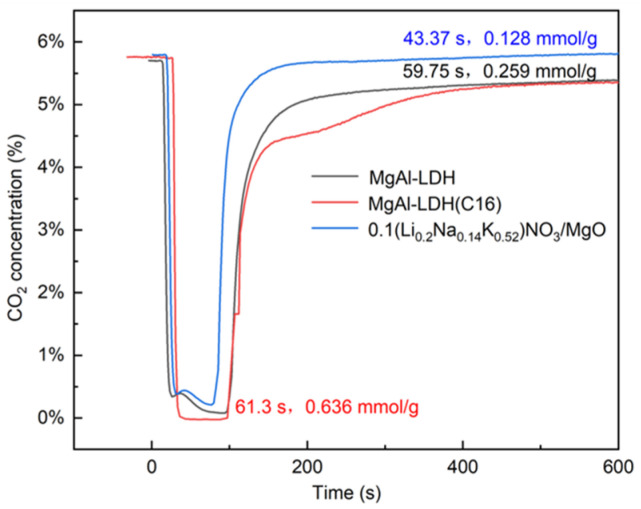
CO_2_ breakthrough curves in sorption capacity measurement using 5.8% CO_2_, and N_2_ balance as gas feed for the adsorption step at 350 °C.

Traditional tests for capturing CO_2_ using solid adsorbents are typically performed using TGA. The CO_2_ atmosphere in TGA is usually maintained at a constant level, and the test results only display the increase in weight of the adsorbent. This approach differs somewhat from actual conditions. However, in practical applications, CO_2_ is predominantly adsorbed in a fixed or fluidized bed, which represents dynamic adsorption. In this work, the dynamic CO_2_ adsorption performances of different adsorbents were studied by measuring the breakthrough curve of the fixed bed. The penetration time of the adsorbent was studied, and the adsorption capacity of the adsorbent was calculated based on the breakthrough curve. The data obtained from TGA were compared with those from the fixed bed. Figure 3 shows the typical breakthrough curve of the LDH and the molten salt-promoted MgO. Under these test conditions, MgO exhibited the shortest penetration time and the lowest adsorption capacity, which is consistent with the results obtained from TGA. The organic anion intercalation LDH demonstrated the highest adsorption capacity and longest penetration time compared to the inorganic ion intercalation, with values of 61.3 s and 0.636 mmol/g, respectively.

### 2.2. The CO_2_ Capture Performance of Layered Double Hydroxide Promoted with K_2_CO_3_

**Figure 4:**
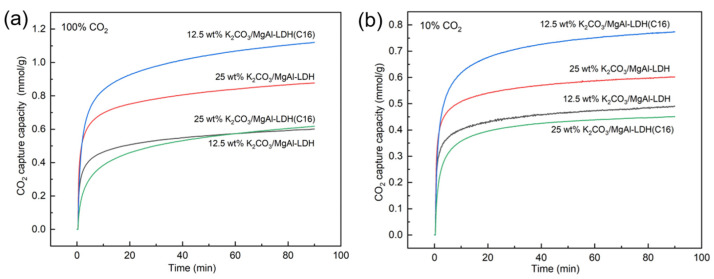
CO_2_ adsorption capacity of LDH promoted with K_2_CO_3_ at (**a**) 100% CO_2_ and (**b**) 10% CO_2_.

Loading LDH-derived adsorbents with K_2_CO_3_ is an effective method for enhancing their adsorption activity. This study also investigated the impact of K_2_CO_3_ loading on the performance of inorganic and organic LDH-derived adsorbents. K_2_CO_3_ loadings of 25 wt% and 12.5 wt% were used to prepare promoted LDH adsorbents, as shown in Figure 4. The adsorption capacity of several samples in 100% CO_2_ concentration was significantly enhanced by K_2_CO_3_ loading, which is consistent with previous studies [39]. To investigate the adsorption performance of the samples under low CO_2_ concentrations, which is more representative of industrial scenarios, this study also tested the adsorption activity of each sample at a 10% CO_2_ concentration. Both loadings of 12.5 wt% K_2_CO_3_ and 25 wt% K_2_CO_3_ demonstrated promotional effects on the LDH. However, the optimal loading capacity varied for different carriers. Specifically, MgAl–LDH reached its optimum loading capacity at 25 wt% K_2_CO_3_, while MgAl–LDH(C16) achieved its best performance at 12.5 wt% K_2_CO_3_. Remarkably, the adsorption capacity of 12.5 wt% K_2_CO_3_/MgAl–LDH(C16) reached 0.8 mmol/g at 10% CO_2_, indicating that the concentration of CO_2_ had minimal impact on the adsorption capacity of LDH. This is in stark contrast to MgO, as the study found that, when MgO was modified with molten salts, it exhibited an adsorption capacity exceeding 10 mmol/g in 100% CO_2_ at 300 °C. However, in a low CO_2_ concentration condition, the adsorption performance significantly declined. For a 10% CO_2_ concentration, its adsorption capacity was less than 0.1 mmol/g [44]. This indicates that LDH-derived adsorbents have more potential for practical applications at low CO_2_ concentrations.

**Figure 5:**
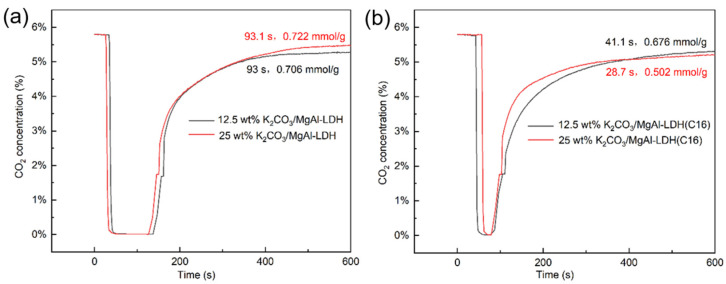
CO_2_ breakthrough curves of (**a**) K_2_CO_3_-promoted MgAl–LDH and (**b**) K_2_CO_3_-promoted MgAl–LDH(C16).

Figure 5 shows typical breakout curves for LDHs with different K_2_CO_3_ loadings. It is evident that the modification of LDH with K_2_CO_3_ significantly enhances its CO_2_ adsorption capacity, corroborating the findings from the TGA results. However, for the inorganic anion-intercalated MgAl–LDH, the penetration time and dynamic adsorption capacity were both improved, to some extent, after K_2_CO_3_ loading. In contrast, the improvement in adsorption capacity was not significant for organic anion-intercalated MgAl–LDH(C16) after K_2_CO_3_ loading. Conversely, the dynamic adsorption capacity of the material with 25 wt% K_2_CO_3_ loading even decreased, which may be due to an excessive K_2_CO_3_ load resulting in pore or surface blockage. Figure S2 explores the breakout curves of 25 wt% K_2_CO_3_/MgAl–LDH and 12.5 wt% K_2_CO_3_/MgAl–LDH(C16) at various gas flow rates. It is observed that as the flow rate increases, the penetration time decreases. This phenomenon can be attributed to the dynamic adsorption process, where a lower gas velocity allows CO_2_ to linger on the adsorbent surface, resulting in a longer penetration time. The nitrogen adsorption–desorption analysis revealed that the specific surface area of both MgAl–LDH and MgAl–LDH(C16) decreased with increasing K_2_CO_3_ load, as shown in Table 2. The sample curves in Figure S3 are consistent with type IV isotherms and H3 hysteresis loops, and it can be judged that samples belong to mesoporous materials according to pore size distribution. In the dynamic adsorption experiments, 25 wt% K_2_CO_3_/MgAl–LDH exhibited the highest CO_2_ adsorption activity, with a maximum adsorption capacity of 0.722 mmol/g and a penetration time of 93.1 s. This result is in contrast to the findings from the TGA, where 12.5 wt% K_2_CO_3_/MgAl–LDH(C16) achieved the highest adsorption capacity over a period of 90 min, as shown in Figure 4. The discrepancy can be explained by the short duration of the dynamic adsorption experiments, where the adsorption rate is the main influencing factor on adsorption activity rather than the saturation adsorption capacity. In comparison, 25 wt% K_2_CO_3_/MgAl–LDH exhibited a faster reaction rate during the initial stages of adsorption.

**Figure 6:**
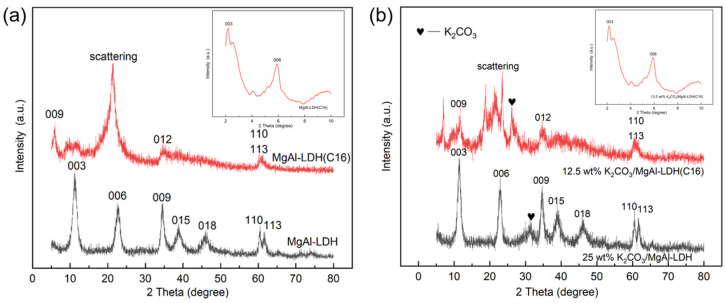
(**a**) MgAl–LDH and MgAl–LDH(C16). (**b**) K_2_CO_3_-promoted MgAl–LDH and MgAl–LDH(C16).

Figure 6 shows the X-ray diffraction (XRD) patterns of LDHs with carbonate anion intercalation and organic multi-carbon chain anion intercalation, as well as with K_2_CO_3_ loadings. The characteristic Bragg reflection of LDH can be clearly observed in all samples, confirming the successful formation of LDH structures. In Figure 6a, for MgAl–LDH and MgAl–LDH(C16), the 003 peak is observed at 11.3° and 2.1°, the 006 peak is observed at 22.6° and 5.8°, the 009 peak is observed at 34.5° and 6.1°, and the 012 peak of MgAl–LDH(C16) is observed at 34.9°; 003, 006, 009, and 012 can be indexed as typical hydrotalc-like structures (JCPDS 15-0087). When carbonate ions are replaced by anions in palmitic acid, the peak value of 003 shifts to a lower value, indicating that the insertion of long carbon chain organic anions increases the interlayer distance of LDH layers. All the above findings confirm the successful synthesis of MgAl–LDH and MgAl–LDH(C16), with MgAl–LDH exhibiting a basal spacing (d_003_) of 8.01 Å and MgAl–LDH(C16) showing a basal spacing (d_003_) of 40.89 Å; this indicates that the interlayer spacing of the material is increased by the intercalation of organic anions. In Figure 6b, it can be found that the typical characteristic peaks of hydrotalc-like compounds still exist before calcination after MgAl–LDH and MgAl–LDH(C16) impregnation of K_2_CO_3_, indicating that the impregnation of K_2_CO_3_ did not destroy the layered structure of the LDH. A series of small peaks between 2θ = 25° and 35° in carbonate sample are typical of K_2_CO_3_ [30]. The basal reflection of LDH is quite wide and not too strong, indicating that the layered structure has low crystallinity and may be disordered. It has been reported that the crystallinity and order of the layered structure of LDH containing organic anions are largely dependent on the properties of the anions [45].

**Figure 7:**
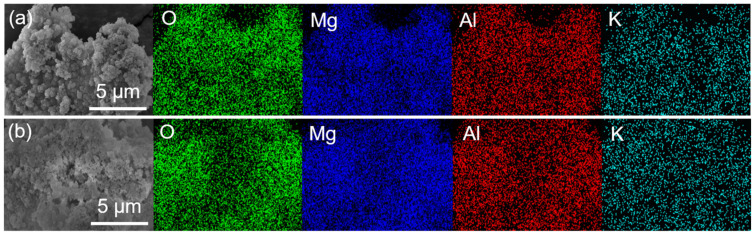
SEM–EDS analysis of (**a**) 25 wt% K_2_CO_3_/MgAl–LDH and (**b**) 12.5 wt% K_2_CO_3_/MgAl–LDH(C16).

**Figure 8:**
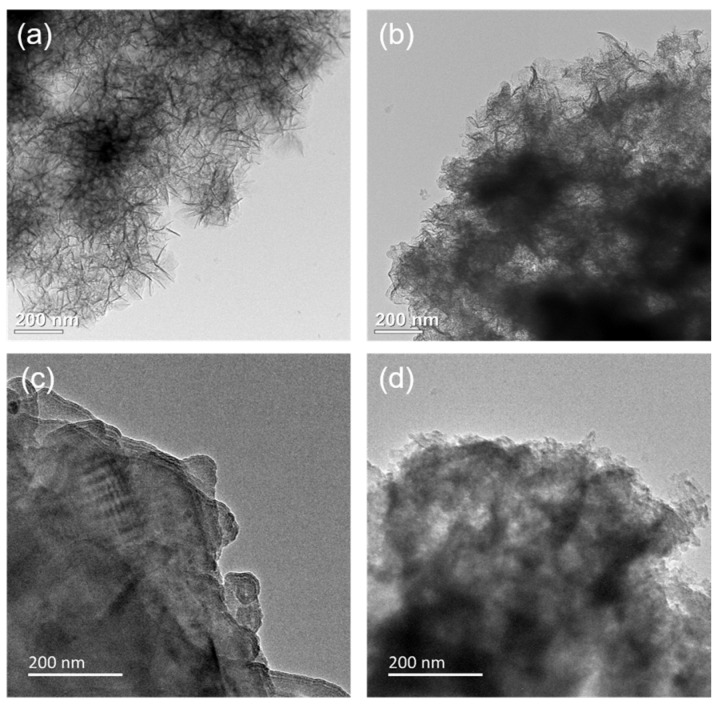
TEM images of adsorbents: (**a**) MgAl–LDH, (**b**) MgAl–LDH(C16), (**c**) 25 wt% K_2_CO_3_/MgAl–LDH, and (**d**) 12.5 wt% K_2_CO_3_/MgAl–LDH(C16) after calcination.

The morphology and microstructure of MgAl–LDH and MgAl–LDH(C16) with different K_2_CO_3_ loadings were observed by SEM, as shown in Figure S4. MgAl–LDH exhibited a lamellar stacking structure, and its basic morphology remains unchanged after loading K_2_CO_3_. After calcination, MgAl–LDH(C16) still maintained a flaky nanoparticle morphology. However, when loaded with 12.5 wt% K_2_CO_3_, it formed a flaky clustered structure, which may have exposed more basic sites on the surface for CO_2_ adsorption. At a loading capacity of 25 wt% K_2_CO_3_, the pores became blocked, due to excessive loading; this correlated with a decrease in the CO_2_ adsorption capacity of the adsorbent at high loadings. After loading K_2_CO_3_, these small particles blocks the pores on the surface of the adsorbent to varying degrees, with greater pore blockage occurring as the loading load increased. Figure 7 presents SEM–EDS analysis of two adsorbents, 25 wt% K_2_CO_3_/MgAl–LDH and 12.5 wt% K_2_CO_3_/MgAl–LDH(C16). The figure illustrates the actual distribution of O, Mg, Al, and K elements on the surface of the adsorbent, showing that the four elements are evenly distributed without agglomeration. The results indicate successful loading of K_2_CO_3_ onto the surface of the LDH without disrupting the nanosheet structure on the adsorbent’s surface. Figure 8 provides TEM images of carbonate and palmitic acid anion-based LDH adsorbents calcined at 400 °C, captured at the same magnification. Both MgAl–LDH and MgAl–LDH(C16) exhibit a rose-like appearance. It is observed that amorphous adsorbents obscure the original brucite-like layers and aggregate within the interlayers; the brucite-like layers of 12.5 wt% K_2_CO_3_/MgAl–LDH(C16) collapsed and broke into smaller-sized amorphous, dispersed with black spots, which are dispersed K elements on the adsorbent’s surface.

### 2.3. Fit Adsorption Process and Compared by Rate Constant

**Figure 9:**
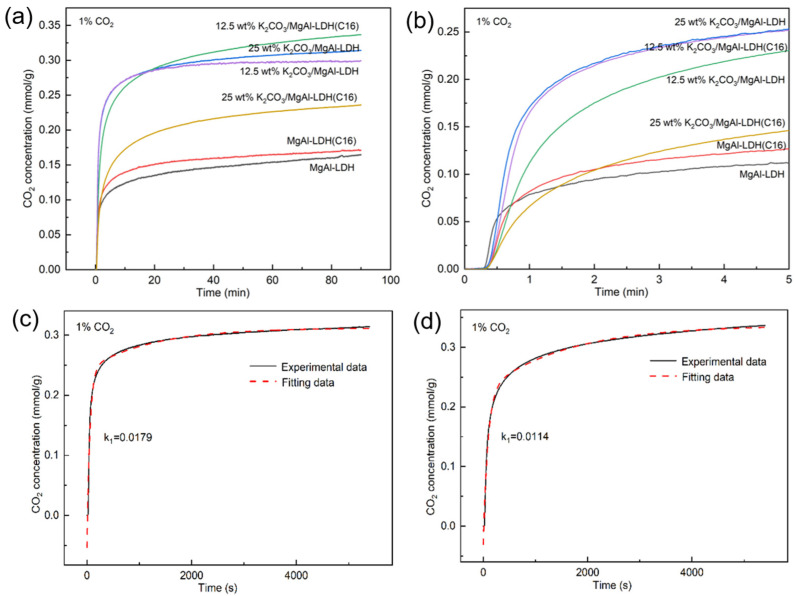
(**a**) CO_2_ adsorption capacity of LDH with different K_2_CO_3_ loads at 1% CO_2_. (**b**) shows the trend in the first five minutes; the dual exponential model was used to fit the adsorption isotherm of (**c**) 25 wt% K_2_CO_3_/MgAl–LDH (**d**) 12.5 wt% K_2_CO_3_/MgAl–LDH(C16).

To investigate the reason why the dynamic adsorption activity of 12.5 wt% K_2_CO_3_/MgAl–LDH(C16) is lower than that of 25 wt% K_2_CO_3_/MgAl–LDH, several LDH samples with different K_2_CO_3_ loads were analyzed, using a TGA under low concentration conditions (1% CO_2_). The results are shown in Figure 9a,b. It can be seen that the adsorption capacity of several samples follows the same trend as that observed for 100% CO_2_ and 10% CO_2_; when the adsorption time reached 90 min, 12.5 wt% K_2_CO_3_/MgAl–LDH(C16) displayed optimal adsorption activity. However, during the initial stage of adsorption, the inorganic MgAl–LDH loaded with K_2_CO_3_ exhibited a faster adsorption rate. It was only after 20 min of reaction time that the adsorption capacity of 12.5 wt% K_2_CO_3_/MgAl–LDH(C16) started to fall behind. When it comes to dynamic adsorption, the process usually only lasts for 1–2 min, and the CO_2_ concentration in the gas decreases to extremely low levels during this time. In such a case, 25 wt% K_2_CO_3_/MgAl–LDH was found to be the most effective in terms of activity.

The adsorption capacity of CO_2_ was drawn according to the kinetic model, the adsorption process was fitted, the dynamic change in absorption during the reaction between the adsorbent and CO_2_ was studied, and the adsorption rate of the adsorbent was further compared by the rate constant [46]. Figure 9c,d shows the dual exponential model fitted by adsorption isotherms of 25 wt% K_2_CO_3_/MgAl–LDH and 12.5 wt% K_2_CO_3_/MgAl–LDH(C16) at 1% CO_2_. The model assumes the CO_2_ adsorption process consists of two stages: chemisorption (k_1_) and mass transfer (k_2_). The former is a rapid reaction stage, while the latter reflects the slow mass transfer of CO_2_ within the adsorbent. The double exponential model can be represented as y = Aexp(−k_1_t) + Bexp(−k_2_t) + C, where y represents the adsorption capacity, t is the adsorption time in seconds, and k_1_ and k_2_ are exponential constants. The kinetic parameters are summarized in Table 3. The experimental data show that 25 wt% K_2_CO_3_/MgAl–LDH has a higher k_1_ value, which means that it has a higher adsorption rate and a higher adsorption amount in a short time. Also, 25 wt% K_2_CO_3_/MgAl–LDH has a higher k_2_ value, which means it has a higher diffusion rate.

The CO_2_–TPD analysis was conducted to determine the number and distribution of basic sites on the adsorbents. Figure S5 displays the CO_2_–TPD characterization results for the four adsorbents. According to the previous reports, the temperature segments of (1) 50–150 °C, (2) 150–400 °C, and (3) >400 °C can be divided into the following three types of basic sites: weakly basic sites (Brønsted hydroxyl groups), moderately basic sites (Lewis acid–base centers), and strongly basic sites (Lewis bases associated with oxygen anions) [7]. From Figure S5, it can be observed that desorption peaks of MgAl–LDH and MgAl–LDH(C16) are concentrated at 100–150 °C and 500–750 °C, respectively. This is due to the desorption of bridging carbonates from weakly alkaline sites and bidentate carbonates from strongly alkaline sites, respectively. After doping carbonate, the peak of the strong alkaline site shifted to a higher temperature. The desorption peaks of 25 wt% K_2_CO_3_/MgAl–LDH and 12.5 wt% K_2_CO_3_/MgAl–LDH(C16) are concentrated at 400–900 °C, and the peaks shifted towards higher temperatures as the loading increased. It is inferred that the doping of K_2_CO_3_ increased the number of strongly alkaline sites, thus promoting CO_2_ adsorption. Figure S6 displays the Fourier Transform Infrared Spectroscopy (FTIR) spectra of LDH and K_2_CO_3_-promoted LDH, allowing for the identification of the chemical components of LDH. The band near 3440 cm^−1^ corresponds to the stretching of -OH, and 1640 cm^−1^ is attributed to the angular deformation vibration of water molecules. The bands near 640 cm^−1^ and 470 cm^−1^ originate from the vibration of the Mg-O and Al-O bonds [13]. Following K_2_CO_3_ promotion, a new peak appeared at 860 cm^−1^, indicating the superposition of different carbides and suggesting that potassium enhances the reaction between the sample and CO_2_.

**Figure 10:**
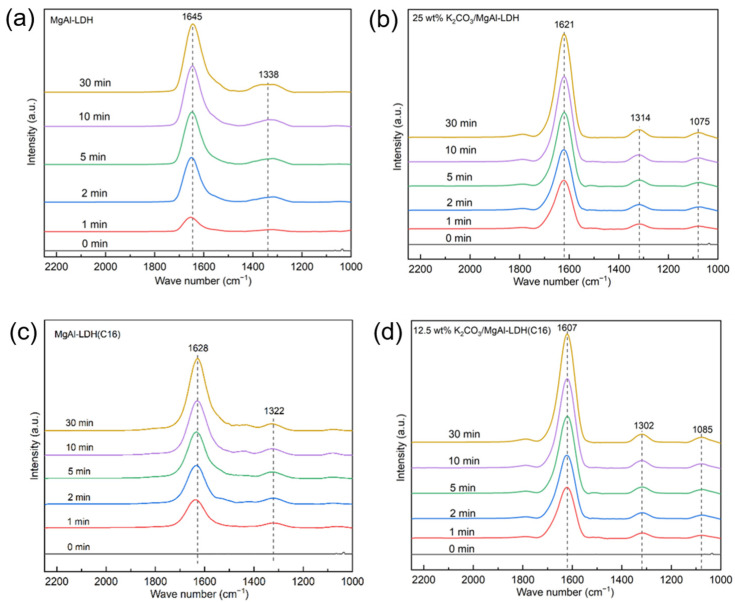
In situ FTIR spectra of (**a**) MgAl–LDH (**b**) 25 wt% K_2_CO_3_/MgAl–LDH (**c**) MgAl–LDH(C16) and (**d**) 12.5 wt% K_2_CO_3_/MgAl–LDH(C16) at 350 °C with different reaction times.

**Figure 11:**
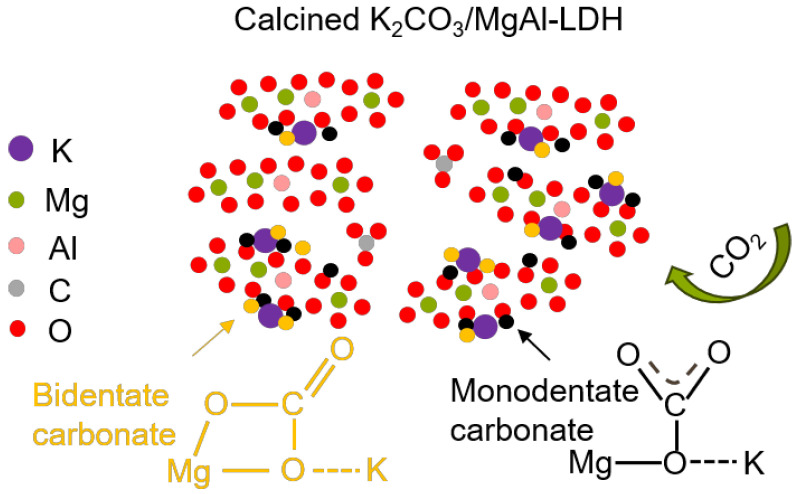
Schematic illustration of the CO_2_ adsorption routes of K_2_CO_3_/MgAl–LDH.

To theoretically analyze the intermediate temperature CO_2_ capture performance of the LDH, in situ FTIR analysis was conducted, as shown in Figure 10. Due to the super-fast adsorption rate in pure CO_2_, a lower partial pressure of CO_2_ (1% CO_2_) was employed. The vibrations near 1600 cm^−1^ and 1300 cm^−1^ in Figure 10 show the v3 asymmetric stretching of the carbonate group. This is attributed to the LDH forming bidentate carbonate when it adsorbs CO_2_. The vibrations near 1080 cm^−1^ in Figure 10b,d indicate the close interaction between the surface carbonate and potassium ions provided by potassium carbonate. In the process of CO_2_ adsorption, a large amount of K_2_CO_3_ participated in the reaction, so that monodentate carbonates and bidentate carbonates were formed on K_2_CO_3_/LDH, as shown in Figure 11 [47], representing an overlap of different carbides and, hence, suggesting that potassium promotes a chemical reaction between the sample and CO_2_. The band CO_2_ at 1080 cm^−1^ becomes slightly Infraredactive upon CO_2_ adsorption for various carbonate species, such as unidentate and bidentate [48]. 

## 3. Materials and Methods

### 3.1. Synthesis of Adsorbents

LDHs with carbonate ion and organic anion intercalation were synthesized via the coprecipitation process. The following is a description of the Mg_3_Al_1_-CO_3_ preparation process: in 100 mL deionized water, 0.075 mol Mg(NO_3_)_2_·6H_2_O and 0.025 mol Al(NO_3_)_3_·9H_2_O were dissolved, and 0.05 mol Na_2_CO_3_ was dissolved in 100 mL deionized water. The metal salt solution and NaOH (4 M) were added to the Na_2_CO_3_ solution drop by drop. The preparation technique for Mg_3_Al_1_-C16 is outlined as follows: 0.025 mol palmitic acid (C16), 0.0375 mol Mg(NO_3_)_2_·6H_2_O, and 0.0125 mol Al(NO_3_)_3_·9H_2_O were dissolved in 150 mL methanol at 80 °C. After all of the ingredients had been dissolved, NaOH (4 M) was added to bring the pH to 10. The resultant mixture was aged for 12 h with constant agitation before being washed with deionized water until the pH reached 7, after which it was dried in an oven at 60 °C.

Mg_3_Al_1_CO_3_–LDH and Mg_3_Al_1_–LDH(C16) promoted by K_2_CO_3_ were produced using a wet impregnation method. The K_2_CO_3_ solution (in water) and LDH suspension (in alcohol) were mixed and thoroughly agitated at 80 °C before drying at 60 °C. In this article, Mg_3_Al_1_CO_3_–LDH and Mg_3_Al_1_–LDH(C16) were impregnated with 12.5 and 25 wt% K_2_CO_3_, respectively.

### 3.2. Characterization of Samples

The materials’ structures were characterized by powder X-ray diffraction (XRD) using Shimadzu XRD-7000 equipment (Kyoto, Japan) in reflection mode, with Cu Kα radiation and a power of 40 kV × 40 mA (λ = 1.542 Å). Diffraction patterns were recorded from 2θ = 5–80°, with a step size of 0.02°. The materials’ morphology was determined using scanning electron microscopy (SEM, Hitachi SU8010, Tokyo, Japan). The functional groups of the LDH were identified using a Fourier transform infrared spectrometer (FT-IR, Spectrum 3, Perkin Elmer, Waltham, MA, USA). BET specific surface area, pore volume, and pore size distribution of the samples were measured using N_2_ sorption/desorption studies (Builder, SSA-7000, Beijing, China). The surface basicity of the LDH was determined using temperature-programmed desorption of carbon dioxide (CO_2_-TPD).

### 3.3. Evaluation of CO_2_ Adsorption Capacity

The CO_2_ adsorption capacity of adsorbents was determined using a thermogravimetric analyzer (TGA, Q50 TA Instrument, New Castle, DE, USA). LDHs were first calcined in a muffle furnace at a specific temperature (400 °C) for 5 h in air before being transported to the TGA analyzer. Prior to CO_2_ adsorption, samples were calcined in situ at 400 °C for 1 h using a high purity N_2_ flow (40 mL/min). The temperature was then reduced to the desired adsorption temperature, and the gas feed was changed from N_2_ to CO_2_, with a steady flow of high purity CO_2_ (1 atm, 40 mL/min), with the CO_2_ adsorption uptake measured for 90 min.

A fixed-bed reactor was utilized to test the adsorbent’s dynamic CO_2_ adsorption capability. The gas volume flow rate was 40 mL/min, and the inlet concentration remained constant. Ar was used as a protective gas, and the temperature was set to 400 °C to activate the adsorbent. The system was then allowed to cool to the temperature required for analysis. The mass flow meter was then set to the desired volume flow rate in order to determine the feedstock gas composition. The gas valve was then switched to the CO_2_ and N_2_ mixture, and the composition of the exit gas was monitored during the adsorption trials. When the composition of the exit gas was similar to that of the feeding gas, the experiment was terminated, and the dynamic adsorption experiment was completed.

## 4. Conclusions

This article presents a systematic study on the dynamic adsorption characteristics of medium-temperature CO_2_ adsorbents based on LDHs. The preparation and comparison of K_2_CO_3_/MgAl–LDH and K_2_CO_3_/MgAl–LDH(C16) for CO_2_ adsorption performance in TGA and fixed-bed experiments were conducted. The results indicate that, in the TGA tests, 12.5 wt% K_2_CO_3_/MgAl–LDH(C16) exhibits higher CO_2_ adsorption activity, reaching a maximum of 1.12 mmol/g. However, the adsorption performance reduced significantly as the CO_2_ concentration decreased, or for the dynamic adsorption process. In the dynamic adsorption process in the fixed-bed test, 25 wt% K_2_CO_3_/MgAl–LDH demonstrated better CO_2_ adsorption activity. In this case, it sustained a breakthrough time of 93.1 s and achieved an adsorption capacity of 0.722 mmol/g. This was mainly due to the faster initial adsorption rate of the inorganic LDH towards low-concentration CO_2_. This also indicates the better application prospects of 25 wt% K_2_CO_3_/MgAl–LDH in practical CO_2_ adsorption and separation processes.

## Figures and Tables

**Table 1:** Specific surface area, pore size, and pore volume of MgAl–LDH, MgAl–LDH(C16), and modified MgO.

Samples	BET SSA (m^2^/g)	BJH Pore Size (nm)	BJH Pore Volume (cm^3^/g)
MgAl–LDH	194.29	22.52	1.09
MgAl–LDH(C16)	122.28	14.12	0.43
0.1(Li_0.2_Na_0.14_K_0.52_)NO_3_/MgO	13.11	61.16	0.2

**Table 2:** Specific surface area, pore size, and pore volume of K_2_CO_3_-promoted LDHs.

Samples	BET SSA (m^2^/g)	BJH Pore Size (nm)	BJH Pore Volume (cm^3^/g)
12.5 wt% K_2_CO_3_/MgAl–LDH	100.59	31.3	0.78
25 wt% K_2_CO_3_/MgAl–LDH	65.95	36.3	0.59
12.5 wt% K_2_CO_3_/MgAl–LDH(C16)	10.59	57.82	0.15
25 wt% K_2_CO_3_/MgAl–LDH(C16)	5.43	73.78	0.1

**Table 3:** Summary of the CO_2_ capture performances of 25 wt% K_2_CO_3_/MgAl–LDH and 12.5 wt% K_2_CO_3_/MgAl–LDH(C16).

Samples	k_1_ (s^−1^)	k_2_ (s^−1^)	R^2^
25 wt% K_2_CO_3_/MgAl–LDH	0.0179	0.00081	0.9886
12.5 wt% K_2_CO_3_/MgAl–LDH(C16)	0.0114	0.00064	0.9954

## Data Availability

Data are contained within the article and Supplementary Materials.

## Supplementary Materials

The following supporting information can be downloaded at: https://www.mdpi.com/article/10.3390/molecules29061192/s1. Figure S1: (a) N_2_ adsorption-desorption isotherms and (b) pore size distribution of MgAl-LDH, MgAl-LDH(C16) and modified MgO. Figure S2: CO_2_ breakthrough curves of (a) 25 wt% K_2_CO_3_/MgAl-LDH and (b) 12.5 wt% K_2_CO_3_/MgAl-LDH(C16) when gas volume flow rate varied. Figure S3: (a) N_2_ adsorption-desorption isotherms and (b) pore size distribution of K_2_CO_3_ promoted LDH. Figure S4: SEM images of 12.5 wt% and 25 wt% K_2_CO_3_/MgAl-LDH (a,b) as well as 12.5 wt% and 25 wt% K_2_CO_3_/MgAl-LDH(C16) (c,d). Figure S5: CO_2_-TPD of four adsorbents. Figure S6: FTIR spectra of (a) 0, 12.5, 25 wt% K_2_CO_3_/MgAl-LDH and (b) 0, 12.5, 25 wt% K_2_CO_3_/MgAl-LDH(C16).
