# Supplementary material for: Dynamic Intermediate-Temperature CO2 Adsorption Performance of K2CO3-Promoted Layered Double Hydroxide-Derived Adsorbents
Source: Molecules. 2024 Mar 7;29(6):1192. doi: 10.3390/molecules29061192 (PMC10975147; doi:10.3390/molecules29061192)
Supplement: Supplementary file 1 [file molecules-29-01192-s001.zip › molecules-2863912-supplementary.pdf]

Supplementary Information

Dynamic Intermediate-Temperature CO_2 Adsorption Performance of K_2CO_3-Promoted Layered Double Hydroxide-Derived Adsorbents

Ruotong Li [1,2], Xixuan Hu [1,2], Liang Huang [1,2,*], Nicholas Mulei Musyoka [3], Tianshan Xue [4] and Qiang Wang [1,2]

1 Engineering Research Center for Water Pollution Source Control & Eco-Remediation, College of Environmental Science and Engineering, Beijing Forestry University, Beijing 100083, China

2 State Key Laboratory of Efficient Production of Forest Resources, Beijing Forestry University, Beijing 100083, China

3 Nottingham Ningbo China Beacons of Excellence Research and Innovation Institute, University of Nottingham Ningbo China, Ningbo 315100, China

4 Institute of Atmospheric Environment, Chinese Research Academy of Environmental Sciences, Beijing 100012, China

*Correspondence: l_huang@bjfu.edu.cn

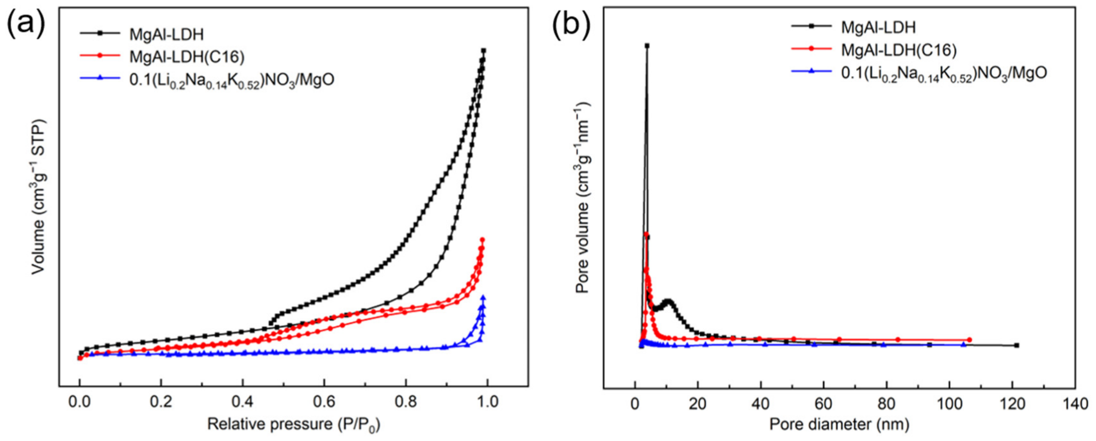

Figure S1. (a) N_2 adsorption-desorption isotherms and (b) pore size distribution of MgAl-LDH, MgAl-LDH(C16) and modified MgO.

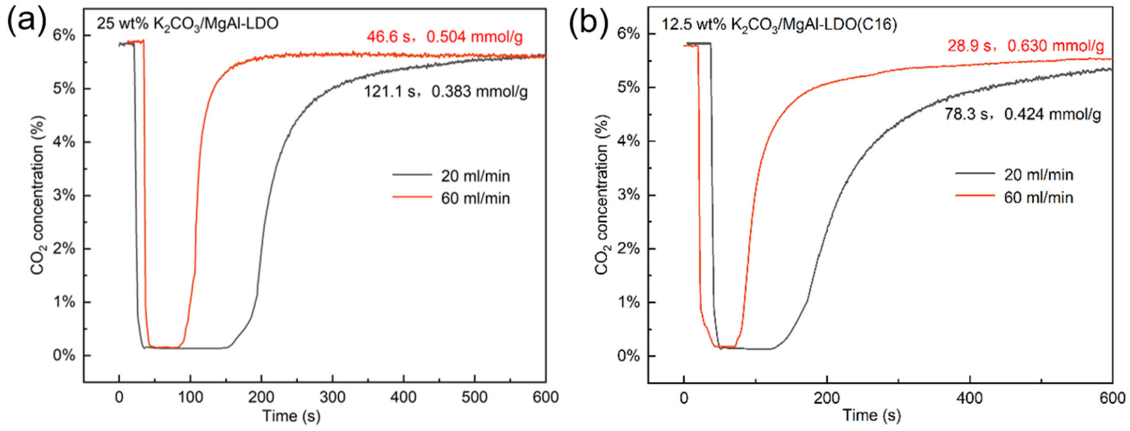

Figure S2. CO_2 breakthrough curves of (a) 25 wt% K_2CO_3/MgAl-LDH and (b) 12.5 wt% K_2CO_3/MgAl-LDH(C16) when gas volume flow rate varied.

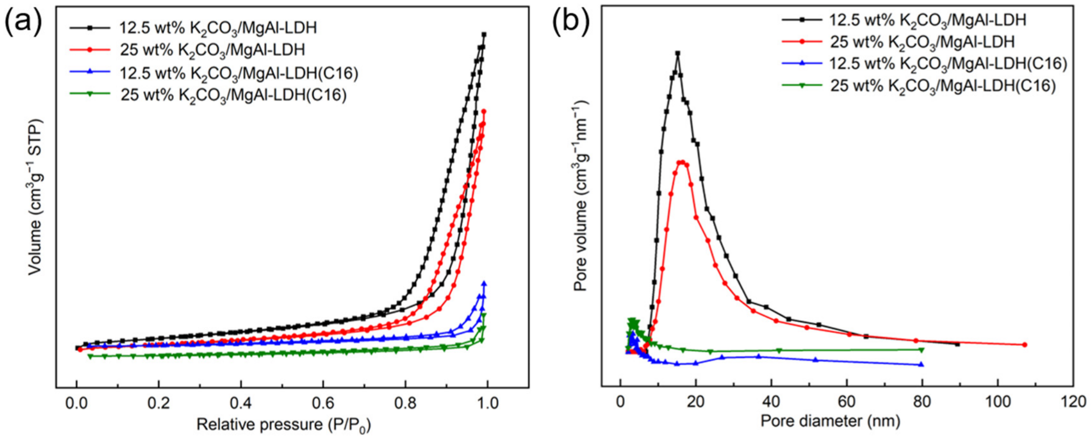

Figure S3. (a) N_2 adsorption-desorption isotherms and (b) pore size distribution of K_2CO_3 promoted LDH.

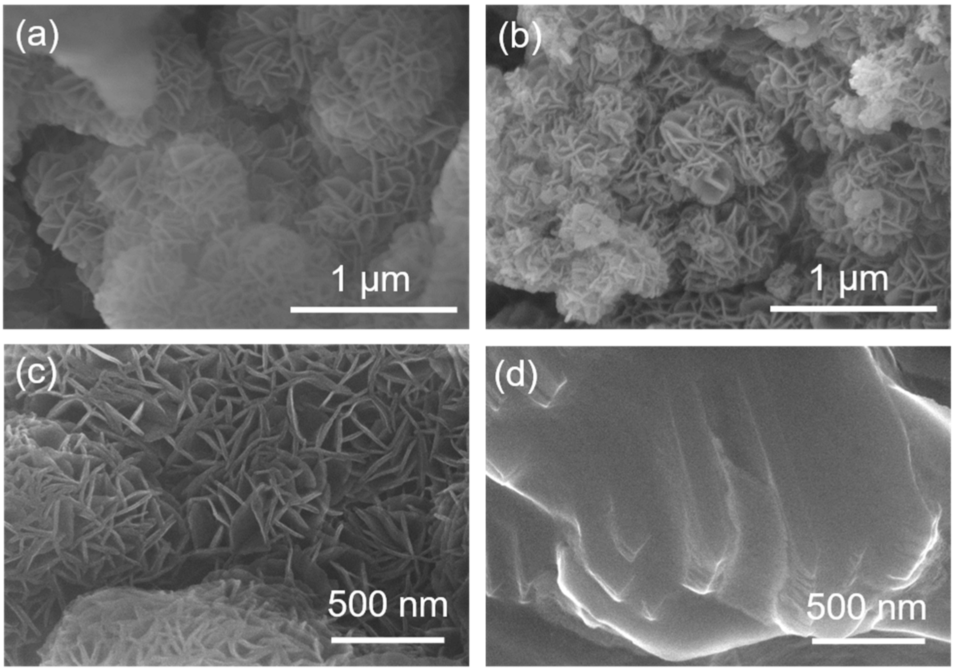

Figure S4. SEM images of 12.5 wt% and 25 wt% K_2CO_3/MgAl-LDH (a,b) as well as 12.5 wt% and 25 wt% K_2CO_3/MgAl-LDH(C16) (c,d).

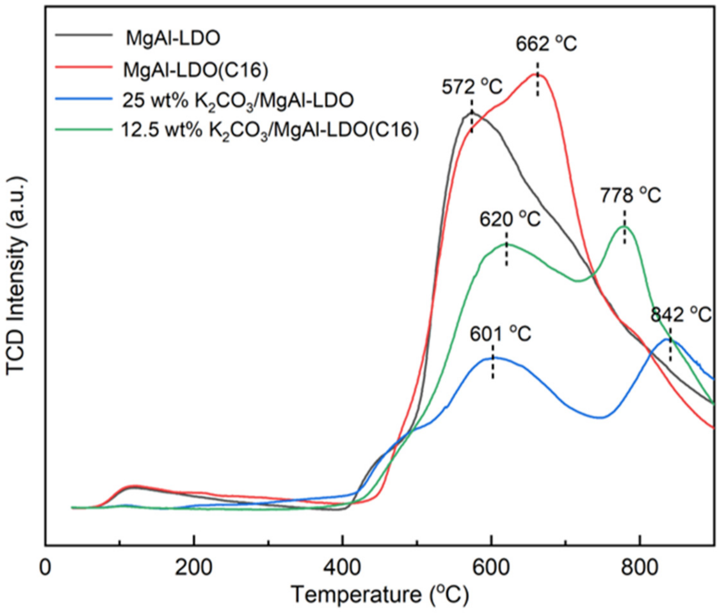

Figure S5. CO_2-TPD of four adsorbents.

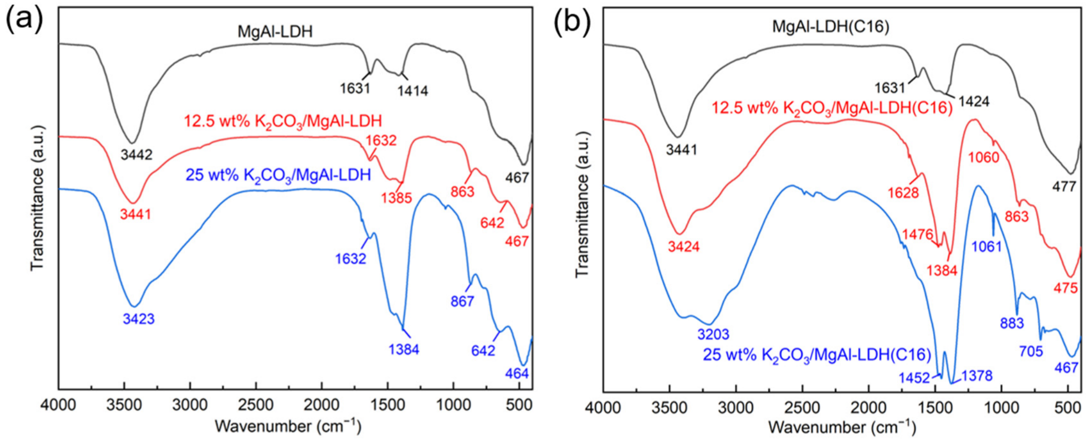

Figure S6. FTIR spectra of (a) 0, 12.5, 25 wt% K_2CO_3/MgAl-LDH and (b) 0, 12.5, 25 wt%K_2CO_3/MgAl-LDH(C16).
